# A human whole-blood model to study the activation of innate immunity system triggered by nanoparticles as a demonstrator for toxicity

**DOI:** 10.1080/14686996.2019.1625721

**Published:** 2019-06-24

**Authors:** Kristina N Ekdahl, Karin Fromell, Camilla Mohlin, Yuji Teramura, Bo Nilsson

**Affiliations:** a Department of Immunology, Genetics and Pathology, Rudbeck Laboratory, Uppsala, Sweden; b Linnaeus Center of Biomaterials Chemistry, Linnaeus University, Kalmar, Sweden; c Department of Bioengineering, The University of Tokyo, Tokyo, Japan

**Keywords:** Coagulation system, complement system, contact/kallikrein system, inflammation, innate immunity, nanoparticles, protein corona, screening, toxicity, whole blood model, 60 New topics / Others, 102 Porous / Nanoporous / Nanostructured materials

## Abstract

In this review article, we focus on activation of the soluble components of the innate immune system triggered by nonbiological compounds and stress variances in activation due to the difference in size between nanoparticles (NPs) and larger particles or bulk material of the same chemical and physical composition. We then discuss the impact of the so-called protein corona which is formed on the surface of NPs when they come in contact with blood or other body fluids. For example, NPs which bind inert proteins, proteins which are prone to activate the contact system (e.g., factor XII), which may lead to clotting and fibrin formation or the complement system (e.g., IgG or C3), which may result in inflammation and vascular damage. Furthermore, we describe a whole blood model which we have developed to monitor activation and interaction between different components of innate immunity: blood protein cascade systems, platelets, leukocytes, cytokine generation, which are induced by NPs. Finally, we describe our own studies on innate immunity system activation induced by three fundamentally different species of NPs (two types of engineered NPs and diesel NPs) as demonstrator of the utility of an initial determination of the composition of the protein corona formed on NPs exposed to ethylenediaminetetraacetic acid (EDTA) plasma and subsequent analysis in our whole blood model.

## Graphical abstract

**Figure:**
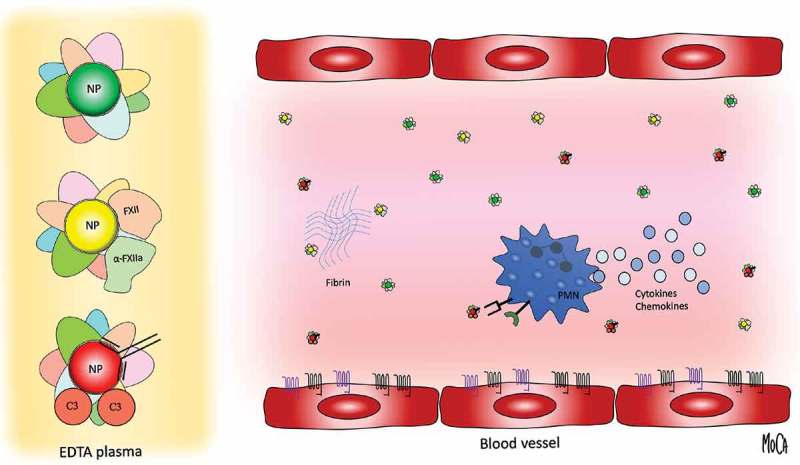

## 1. Introduction

### 1.1. Definition of nanoparticles (NPs) and presentation of the problem

Typically, the term ‘nanoparticles’ (NPs) or ultrafine particles refer to particles with a size up to 100 nm, but in real life there is a sliding scale between small and larger particles both those which are manufactured or are generated by environmental processes (see below) where the size is significant for biological response. Also, in situations where the size of NPs has been carefully evaluated after manufacture, they will inevitably form agglomerates of various size when exposed to different media such as saline, buffers, culture media, or blood plasma or serum [1], further stressing the difficulty to define the particles sizes.

The miniscule size of NPs allows for single or multi-agglomerate NP to enter the body. The main potential routes of NP exposure are inhalation, and oral and dermal exposure through pharmaceutical and food products containing NP additives or NPs present in the environment. The rationale for applying nanotechnological approaches is based on the unique properties associated with nanoscale materials: their different physicochemical characteristics when compared to the same materials in bulk form and their large area-to-mass ratio, which results in a potentially enhanced chemical reactivity with their surroundings. Because of their widespread use, it has become important to evaluate their possible biological toxic effects. Concerns have been raised about associated health risks in the general population at the intended or unintended usage of NPs as well as to exposed workers during the manufacture of industrial NPs [2]. Another general conclusion that can be drawn is that further surface modifications are necessary in order to improve the biocompatibility of NPs intended for therapeutic use. Consequently, there is a need for rapid and simple *in vitro* screening techniques to evaluate the impact of modifications, and determination of protein coronas (see below) on NPs has been put forward as the technique of choice in many studies.

### 1.2. Examples of human exposure to NPs and routes of entry

Human beings are continuously exposed to NPs either in deliberate or cultural applications or by unintentional environmental exposure. The main potential routes of NP exposure are inhalation, and oral and dermal exposure, with some examples presented here:

- NPs are an integral part of food supplement and dental applications [3]. Consequently, they will enter the body via the gastrointestinal channel, as will NPs which are present in toothpaste which may get swallowed.
- NPs present in cosmetics and lotions, obviously will be applied topically, while NPs in tattoo ink will be presented intradermally [4,5].
- Deliberate exposure to NPs takes place in different medical applications where they act as vehicles for drug delivery, as contrast media, etc., and in these applications, they will be administered, e.g., either intravenously, intrathecally or intradermally [6].
- Traffic generated NPs such as present in vehicle exhaust (e.g., diesel particles) or metal NPs from such as wearing of brakes and other parts of vehicles will all be inhaled [7]. The same rationale is true for NPs generated in the industry during the manufacturing of nanomaterials.
- Cooking over an open fire is among the largest environmental health issues globally today and generates large amounts of smoke particles typically in the range of 10–500 nm [8,9]. In addition, other cultural applications such as cigarette smoking [10] or the religious use of incense [11], are also situations which lead to inhalation of NPs by individuals who are exposed to the smoke.
- Finally, environmental disasters such as wildfires and volcanos eruptions generate NPs as well as larger particles which will be inhaled. In the case of volcanic ash, the NPs can act as a carrier for toxic elements on the global scale [12,13].

Exposure to natural or environmental NPs (IV-VI) is difficult to avoid, while exposure to manufactured NPs (I-III) is (at least theoretically) possible to control. The main focus of this article is the response to engineered NPs.

Independent of the source, NPs are generally more reactive and toxic than larger particles of the same material [14] and particulate matter [15,16]. Previous studies of particle toxicity in a lung model system have demonstrated that metal particles in the nano-range generally are more reactive and toxic compared to larger particles [17–19].

It has also been shown that toxicity of particles is dependent on other particle properties such as particle solubility, wettability, surface charge, rigidity and surface oxidation. However, it is complicated to evaluate the influence of a specific physicochemical property on the biological response, since most particles are presenting several different surface properties, that are also affected by the environment in which they are suspended. When the NP surface comes in contact with blood or other body fluids, an initial layer of proteins is immediately adsorbed to the surface, which will constitute a new interface to the blood, cells, and tissues. The composition and confirmation of this protein layer largely affects the activation of the complement and the coagulation systems.

## 2. The innate immune system

### 2.1. Immune system defense lines

Schematically, the human immune system can be separated into innate immunity and acquired immunity. Innate immunity of one kind or other exists in all multicellular organisms, including plants. In contrast, acquired immunity has characteristics such as immunological memory and extremely high specificity and uses lymphocytes (B and T cells) as well as antibodies as effector systems. This form of immunity developed late in evolution and it has been estimated that only ≈5% of the world’s now living species has this line of defense [20,21].

Typically, innate immunity defense is triggered instantly when encountering an antigen. It is able to differentiate between own (autologous tissue) and foreign substances, such as bacteria or biomaterials including NPs and larger particles, but it lacks immunological memory. This means that the effect will neither improve nor deteriorate upon repeated exposure to the causative agent. Inflammation is the central mechanism of action that is driven by activation of the blood cascade system: the complement system, the contact/kallikrein, and coagulation systems and the fibrinolytic system. Each one of the cascade systems consists of a large number of proteins that can be found in most body fluids, where they circulate in non-activated form (as zymogens or pro-enzymes) as well as of cell-bound proteins that function as receptors and control proteins. A common feature for all cascade systems is that in order to accomplish full function they require 1) recognition, 2) initiation, and 3) amplification.

**Figure 1.**
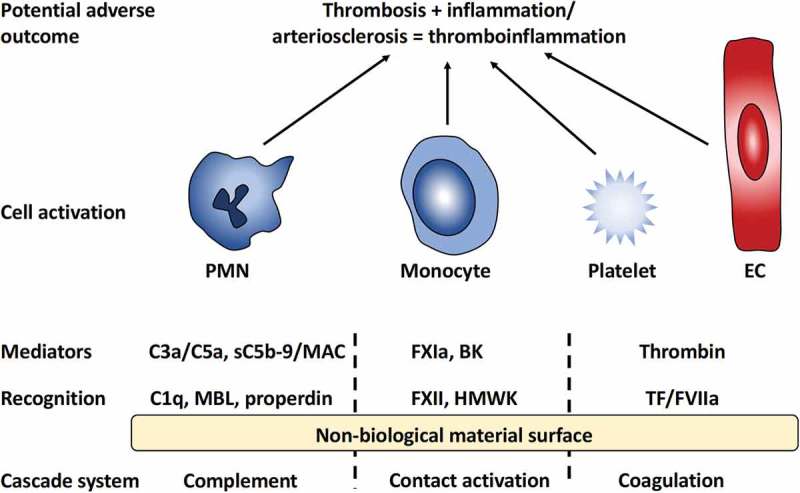
Overview of innate immunity reactions initiated by the interaction between blood and a non-biological material surface. Recognition molecules of the different cascade systems target non-self-structures on the surface. C1q, mannan-binding lectin (MBL), and properdin trigger the complement system generating the anaphylatoxins C3a and C5a as well as the lytic sC5b-9 and membrane attack complex (MAC). Activation of the contact system is triggered by Factor XII (FXII) and high molecular weight kininogen (HMWK), leading to the activation of FXI and generation of the potent anaphylatoxin bradykinin (BK); binding of FVII and tissue factor (TF) of the coagulation system ultimately leads to the generation of thrombin from prothrombin. Collectively, these mediators generated by the cascade systems trigger the activation of polymorphonuclear leukocytes (PMNs), monocytes, platelets, and endothelial cells (ECs) that may lead to inflammatory and thrombotic reactions, i.e., a thrombo-inflammation, that may seriously harm the patient.

The generated activation products in turn trigger activation of platelets and different populations of leukocytes (white blood cells) such as polymorphic nuclear leukocytes (PMNs) and monocytes (Figure 1).

It should be emphasized that under physiological conditions, plasma proteins are found not only in the bloodstream but also in other tissues due to capillary leakage. During inflammation, there is increased capillary permeability, induced primarily by bradykinin (BK; see below) with the consequence that the interstitial concentration of plasma proteins approaches that which is found in the blood. General features of the cascade systems within the innate immune as well as their interactions are discussed in detail in [22].

### 2.2. The complement system

The primary function of the complement system is to act as a purging system that removes agents that do not belong in the body such as pathogens, antigen-antibody complexes, and apoptotic cells. It consists of ≈50 proteins that are soluble in blood plasma or expressed on cells where they act as receptors and regulators that protect autologous tissue against complement attacks. Schematically, the system is divided into three activation pathways known as the classical, the alternative, and the lectin pathway. Each pathway is activated by proteins which recognize structures (often carbohydrates) expressed on pathogens, apoptotic or ischemic cells, but not on healthy autologous cells. This leads to the formation of two different enzyme complexes, so-called C3 convertases, both mediating proteolytic cleavage and activation of C3, which is the central and most abundant component of the system. It is an asymmetric cleavage that gives rise to the smaller anaphylatoxin C3a and the larger fragment C3b that can bind covalently to amino acids or carbohydrates on the target particle and thereby facilitates its phagocytosis. Further activation of the complement system leads to cleavage of C5 to the anaphylatoxin C5a (even more potent than C3a), and C5b which is the basis for the formation of the multi-molecular complex C5b-9 or membrane attack complex (MAC) which can perforate the membrane on sensitive cells or pathogens thereby destroying them. C5b-9 also exists in a soluble form (sC5b-9) that can activate endothelial cells (ECs). The main function of the anaphylatoxins is to recruit and activate PMNs and monocytes and thereby prepare them to perform efficient phagocytosis.

Autologous cells, but not pathogens or biomaterial or other non-biological surfaces, are protected against complement attack by a large number of membrane-bound regulatory proteins, e.g., decay accelerating factor (DAF), membrane co-factor protein (MCP) and complement receptor 1 (CR1). The protection can also be ‘topped up’ by recruitment of other protective proteins such as factor H and C4b-binding protein (C4BP) from the plasma. This binding is due to their affinity for heparan sulfate and other polymeric carbohydrates which are components of the glycocalyx which covers the ECs. Another important regulator is C1 inhibitor (C1INH) that acts in the plasma where it inhibits proteolytic enzymes which are generated early in the complement cascade (prior to activation of C3) as well as proteases within the contact system (see below).

### 2.3. Contact, kallikrein/bradykinin, and coagulation systems

The plasma contact system is linked to both the coagulation system and the kallikrein/bradykinin system: its primary function is to maintain hemostasis, but it also has an important role in inflammation. Factor XII (FXII) is the primary recognition molecule within the contact system and in particular (but not exclusively) it binds to various negatively charged substances such as heparin, lipopolysaccharide (LPS), and biomaterials (including NPs) which leads to its autoactivation to FXIIa. FXIIa, an active protease which can activate prekallikrein to kallikrein which then releases the highly potent inflammatory mediator BK from high molecular weight kininogen (HMWK). Alternatively, FXIIa can start the intrinsic pathway of coagulation by cleaving FXI to FXIa which then initiates the generation of FXa, thrombin and subsequent fibrin formation and clotting.

The extrinsic pathway of coagulation is initiated when tissue factor (TF), which is not normally present in the bloodstream in active form, is exposed in the blood from the extracellular matrix during vascular injury, or exposed on activated monocytes. TF acts as a receptor for FVIIa (always present in trace amounts in the blood), which can then activate FIX, FX, and prothrombin (to thrombin).

Like the complement system, the contact, kallikrein/bradykinin, and coagulation systems are under strict control *in vivo*. Antithrombin (AT) is the most important inhibitor that inactivates, in particular, FXa, FIXa, FVIIa, and thrombin. The effect of AT is increased dramatically in the presence of heparin or heparan sulfate (see below). FXIIa and FXIa are primarily inhibited by C1INH and to a lower degree of AT.

### 2.4. Protective role of the endothelium

An intact, undamaged endothelium is a blood-compatible surface since it is normally protected by the glycocalyx consisting of a layer of proteoglycans, including heparan sulfate. ECs with an intact glycocalyx are both anti-inflammatory and anti-thrombotic, due to the fact that various inhibitory proteins present in the plasma have an affinity for heparan sulfate and thereby bind into the cell surface and protect it, together with existing cell-bound inhibitors. This applies to the complement inhibitors factor H, C4BP, and C1INH (all mentioned above) as well as the coagulation inhibitors AT, protein C and tissue factor pathway inhibitor (TFPI). Taken together, this means that on an intact endothelium there is negligible deposition of proteins that can act as ligands for leukocytes or platelets.

During an intravascular inflammation, the endothelium may be activated indirectly by cytokines (e.g., tumor necrosis factor) released from leukocytes which have been activated by C5a or other agents, or directly by sublytic concentrations of MAC, and switches to a pro-inflammatory and pro-thrombotic state [23]. The inflamed ECs produce enzymes (heparanases and metalloproteases) that destroy the glycocalyx and expose receptors such as E-selectin and various adhesion proteins, e.g., intracellular cell adhesion molecule 1 (ICAM1) and vascular cell adhesion molecule 1 (VCAM1). These and other proteins contribute to the binding of activated leukocytes (mainly PMNs) and platelets, which in turn leads to further activation and destruction of the endothelium.

## 3. Innate immunity activation by artificial surfaces

### 3.1. Innate immunity activation on biomaterial surfaces

Non-biological materials, which come in contact with blood or blood-plasma get covered by a layer of proteins within seconds of exposure. This protein film is approximately 8 nm thick which corresponds to a monolayer of the most abundant proteins [24,25]. The composition, conformation, and amount of individual proteins are affected by the material’s chemical and physical properties such as hydrophobicity/hydrophilicity, charge (zeta potential), topography, etc. [25–27].

Examples of plasma proteins, which preferentially adsorb to material surfaces in altered or denatured conformation include C3 and IgG (when non-antigen bound), which are then able to activate complement via the alternative pathway and classical pathway, respectively [28,29]. C3 deposited and activated to C3b and iC3b function as ligands to PMN and monocytes which thereby bind into the activating surface. Both of these cell types have receptors for bound C3 fragments: CR3 and CR4 (CD11b/CD18 and CD11c/CD18, respectively) and the cells are further stimulated via these receptors.

Other examples of plasma proteins, which are prone to bind to non-biological surfaces include vitronectin, fibronectin, and fibrinogen, which act as mediators for platelet adhesion, thereby mediating thrombus formation [30]. In the case of fibrinogen, a causal relation between its conformational alteration and the ability to function as a ligand for platelets has been demonstrated [31]. Furthermore, adsorbed von Willebrand factor (vWF) may also act as a ligand for platelets together with fibrinogen that binds via the receptor glycoprotein IIb/IIIa (GPIIb/IIIa; CD41/CD61) and thereby initiate coagulation.

Another interesting example is FXII of the contact system, which becomes auto-activated to α-FXIIa when adsorbed to surfaces, which often but not exclusively are negatively charged. In studies performed using systems of purified proteins, IgG, IgM and human serum albumin (HSA) have all been demonstrated to potentiate the activation of FXII [32,33]. α-FXIIa initiates the intrinsic pathway of coagulation leading to thrombin generation and subsequent fibrin formation [34], or the kallikrein/kinin pathway, which results in the generation of the potent pro-inflammatory and – angiogenetic peptide BK (see below) [35]

The adsorbed proteins also include specific recognition molecules from the different cascade system of the blood (Figure 1): C1q, mannan-binding lectin (MBL) and properdin within the complement system, belonging to the classical pathway, the lectin pathway, respectively, the alternative pathway, FXII (as already mentioned) and HMWK of the contact activation system, and TF and FVII within the coagulation system. These recognition molecules then trigger activation of the respective cascade systems, which leads to the generation of soluble mediators (signal substances) capable of binding to receptors on different types of leukocytes (primarily PMNs and monocytes), platelets, and ECs. This induces activation of these cells and causes inflammation and/or thrombosis (= thromboinflammation) since there is a substantial amount of cross-talk between these systems as discussed in [36].

Some important examples of mediators are the complement-generated anaphylatoxins C3a and C5a that are potent activators of PMN and monocytes and the complex sC5b-9 that can induce platelet activation, and MAC that can induce IL-1beta release, and expression of TF on ECs [37,38].

Thrombin, which is generated from prothrombin at the last stage of the coagulation cascade, drives cellular activation via protease-activated receptors (PAR) 1, 3 and 4 which are expressed on platelets, leukocytes, smooth muscle cells, and ECs. This results in, e.g., the upregulation of cell adhesion molecules on these cells and the release of pro-inflammatory cytokines and reactive oxygen compounds, which overall promotes atherogenesis. BK is generated within the contact system by kallikrein which digests HMWK and thereby releases this nine-amino acid residue long peptide. The activity of BK is mediated via bradykinin receptor (BKR) 1 and 2. BKR2 is expressed constitutively on ECs, smooth muscle cells and leukocytes such as PMNs and lymphocytes. BK mediates inflammation and anaphylactoid reactions via BKR2, leading to chemotaxis (recruitment of leukocytes), increased vascular permeability, smooth muscle cell contraction, and pain. Over a longer time period, BK also leads to increased angiogenesis, thereby representing a potential link to cancer. Under physiological conditions, BKR1 shows low expression, but it is upregulated under inflammatory conditions and induces, e.g., further activation of ECs and PMNs which induce the PMNs to bind to the endothelial surface, and such adhesion is a major feature in artherogenesis.

### 3.2. Innate immunity activation by NPs

It needs to be taken into consideration that NPs will behave differently compared to larger particles or bulk materials and affect biological functions and trigger toxic reactions differently. Some examples are that

1. Unlike bulk materials, NP due to their size have the potential to penetrate into the body by inhalation, absorption through the skin or digestive tract, injection, and absorption or implantation for drugs delivery systems, thereby potentially giving rise to adverse effects [39] and references therein.
2. NPs large surface area to the unit mass/volume also make them much more reactive compared to larger sized counterparts or planar surfaces and may therefore be expected to lead to higher cascade system activation, given identical surface chemistry.
3. The conditions for activation of the cascade system differ significantly on NP compared to material surfaces in that the steric conditions are totally dissimilar. The reactions occurring during the activation of the cascade systems depend on conformational changes and protein–protein interactions.
  - One typical example is C1q, the protein within the complement system which binds to IgG and needs to bind at least two closely located IgG molecules in order to change its conformation, bind and activate C1r (the next component in the classical pathway).
  - In contrast, a regulator such as factor H needs adjacent heparan sulfate or other polysaccharide molecules in order to bind and regulate complement on surfaces [40].
  - Furthermore, the nano-scale curvature (reflecting the size) of NPs has been demonstrated to affect both complement and contact system activation. For example, it has been demonstrated that complement activation was significantly attenuated on a nanostructured gold surface as compared to a nonstructured surface [41]. Likewise, NPs with higher curvature (smaller size) did not denature FXII to the same extent as larger NPs with lower curvature [42]. Since conformational changes in FXII lead to its activation, contact system activation is higher on the larger NPs, given identical chemistry and z-potential [43].

In summary, the steric conditions/radius of curvature may lead to both inactivation and activation and may affect individual parts of the innate immune system differently.

### 3.3. Protein corona formation on NPs

Mapping of plasma protein deposition on NPs exposed to blood plasma (forming a so-called ‘protein corona’) is an emerging approach with the aim of predicting future biological behavior, e.g. [42,44–46]. For example, studies of the biomolecular coronas on iron oxide NPs have focused on the magnetic materials, magnetite, and maghemite, used in bioimaging applications, the so-called superparamagnetic iron oxide nanoparticles (SPIONs) [47,48].

However, it must be taken in account that the composition of a protein corona is dependent on a number of factors not immediately related to the surface chemistry of the NP (Table 1). These include the solvent (e.g., H_2_O, 0.9% NaCl or PBS) which the NPs have been immersed in and which will affect its zeta potential, the anticoagulant used to prepare plasma (EDTA or citrate) and the species of the blood donor. It also appears that the composition and size of the corona are very much influenced by the manufacturing conditions and surface modification of the particles, as well as their size [49–51].

**Table 1.** Examples of protein corona determinations. MALDI-TOF analysis of selected NPs equilibrated in H_2_O, 0.9% NaCl or phosphate buffered saline (PBS) and then incubated in plasma or serum. The proteins are ranked according to the protein score.

	Particle (solvent)	zeta-potential	Plasma/serum (species)	Protein score	Coagulation/ contact system	Comple-ment	Apolipo-proteins	Protein score albumin
[56]	TiO_2_ (PBS)	+24 (mV)	EDTA-plasma (human)	**Top 5** **6–20**	4 4	0 8	0 1	3
[57]	Fe_2_O_3_ (PBS)	−22 (mV)	EDTA-plasma (human)	**Top 5** **6–20**	1 3	0 6	3 2	2
[57]	Fe_2_O_3_ (0.9% NaCl)	+8.8 (mV)	EDTA-plasma (human)	**Top 5** **6–20**	1 4	0 4	3 1	4
[59]	Fe_3_O_4_ (H_2_O)	−23.4 (mV)	EDTA-plasma (human)	**Top 5** **6–20**	0 3	0 3	4 2	2
[60]	Fe_3_O_4_ (H_2_O)	+3.9 (mV)	citrate-plasma (human)	**Top 5** **6–20**	3 0	0 2	0 3	1
[49]	Fe_3_O_4_ (PBS)	−19.4 (mV)	citrate-plasma(?) (human)	**Top 5** **6–20**	0 1	2 3	2 2	32
[51]	SPIONS (0.9% NaCl)	+13 (mV)	Serum (rat)	**Top 5** **6–17**	0 4	0 2	2 0	15
[53]	SPIONS (0.9% NaCl ?)	+16.7 (mV)	Serum (fetal bovine)	**Top 5** **6–16**	0 2	0 2	0 1	1

Another important concern is how to analyze the protein corona. With some methods, the adsorbed proteins are analyzed directly at the NP surface, which means that it primarily is the outermost layer that will be detectable. Other techniques require desorption of the protein layer before analysis. The difficulty is then to ensure that it really is a representative population of the protein corona that is desorbed. Therefore, a suitable approach to avoid methodological bias is to employ more than one method to determine the protein profile at the NPs.

Furthermore, many such studies of protein corona formation have been performed using artificial systems consisting of serum (i.e., the end product after blood coagulation), which is never present *in vivo*. In particular, it is not possible to evaluate studies in cell culture in which fetal bovine serum (FBS) is the source of corona formation on NPs, since the composition of plasma proteins is totally different than that in adult individuals, including an almost total lack of immunoglobulins and complement proteins [52]. Consequently, in situations in which the aim is to study NP reactions in humans, such experimental models are not suitable. However, both *in vitro* studies using various cells (mainly immortalized cell lines) in culture, e.g. [49,50,53,54], and *ex situ* studies in rat models [51] have been reported. If the aim of a determination is to provide a model of the corona which may be formed on NPs under these experimental systems it might be acceptable to use serum from the relevant sources, i.e., fetal calves in the case of cell culture, and rats for such *in vivo* or *ex vivo* studies.

In our laboratory, we instead determine the composition of the corona after incubating the NPs in human EDTA-plasma followed by matrix-assisted laser desorption/ionization time-of flight (MALDI-TOF) measurements. EDTA was chosen instead of other anticoagulants, to ascertain that any detected proteins were passively adsorbed and not accomplished by cascade system activation on the particles since EDTA irreversibly binds both Ca^2+^ and Mg^2+^, thereby inhibiting both the coagulation and the complement systems [55]. The detected proteins include contact system and coagulation proteins, apolipoproteins, serine protease inhibitors (serpins) and complement proteins [56,57].

In contrast, if NP coronas are determined after incubation in citrate-anticoagulated plasma (which is not fully inhibited regarding complement activation) they will include substantial amounts of complement proteins deposited by proteolytic activation, such as C3 and C4. It should also be noted that the presence of EDTA does not affect the contact activation system since its activation is independent of divalent cations. Therefore, it is of utmost importance that samples are treated very carefully prior to analysis, i.e., frozen to at least −70°C, and thawed rapidly at 37°C and transferred to ice prior analysis. This procedure is particularly important regarding the analysis of soluble cascade system activation markers (see below) but also regarding protein corona determination [55].

## 4. Evaluating NP toxicity in vitro

### 4.1. A whole blood test system for evaluating NP toxicity

Serum is frequently used for biocompatibility screening. However, serum is obtained from clotted blood where all coagulation proteins and all cells of the blood have been consumed. Consequently, it is totally impossible to monitor crosstalk between cascade system within innate immunity and the resulting communication with different blood cells [22,55].

Animal models can only be used in a limited number of test applications and are subject to ethical concerns, and the interpretation of experiments in animals is also distorted by the species differences [55].

**Figure 2.**
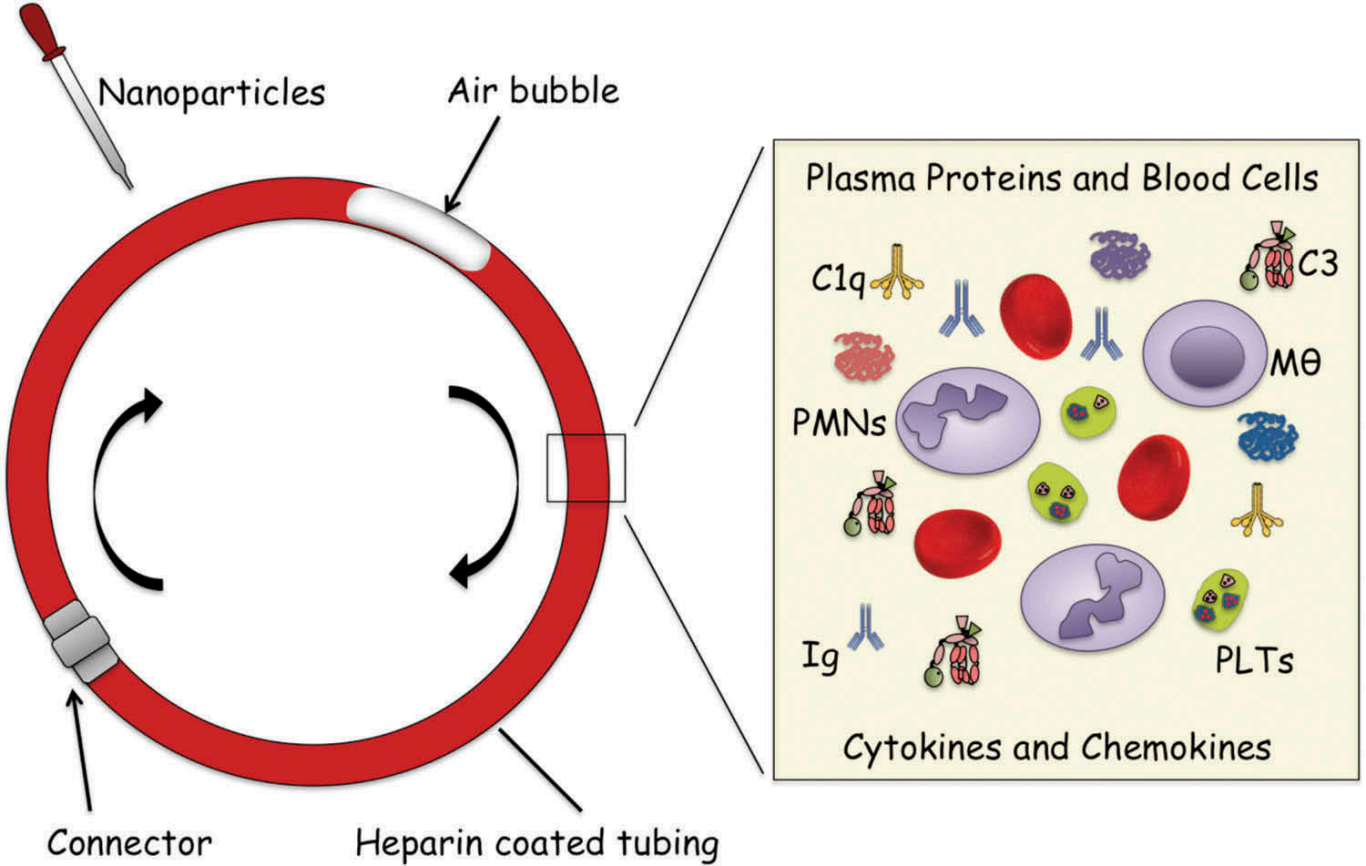
Blood loop model for assessment of activation of innate immunity systems and blood cells induced by nanoparticles (NPs). The model consists of tubing (internal diameter of 4 mm, length of 20 cm) that is covalently coated with heparin. Human blood, freshly drawn and without anticoagulants (2.0 mL) is added together with NPs in final concentration of ranging from 10 ng/mL to 5 µg/mL blood. Thereafter, the tubing is closed to form loops using connectors of stainless steel coated with immobilized heparin and rotated vertically for 60 min at 20 rpm in a 37°C water bath. After or during incubation, aliquots of blood are removed and analyzed for platelet counts (using a cell counter), leukocyte activation (by FACS analysis), and fluid phase markers resulting from activation of the complement, coagulation, contact/kallikrein systems, and release of cytokines and chemokines using various multiplex assays (see text for details). It should be noted that it is not possible to isolate NPs for corona determination after incubation in whole blood since they will adhere to or be phagocyted by leukocytes [61]. An extensive description of the experimental protocol is found in ref [57] and the figure is reproduced with permission from the publisher.

As an alternative we have constructed a novel and highly sensitive loop model using human non-anticoagulated blood which is rotated in heparin-coated tubing in order to enable simultaneous monitoring of NP-induced cascade system activation, plasma system analysis, cell phenotypes, secreted proteins, chemokines and cytokines, as well as the cross-talk between the systems (Figure 2) [56,57]. Fresh human blood containing the NPs is added to tubing which are then closed to loops and rotated at 37°C in a water bath or a cabinet, typically for up to 1 h. Surface-heparinized equipment is used to minimize surface-dependent activation to ascertain that the detected effect is induced by the test substance of interest, and not by the tubing surface. It is possible to totally avoid soluble anticoagulant if surface-heparinized equipment is used for collecting and dispersing the blood.

After incubation, the blood is removed from the tubing into EDTA-containing tubes in order to interrupt further activation of the coagulation and complement system (but not of the contact system since it is not dependent on divalent cations). At this stage, platelets are counted (as an indirect measure of coagulation activation) and leukocytes may be stained for different activation markers for further analysis using flow cytometry. Then, the plasma is separated by centrifugation and used for the analysis of activation products in the fluid phase using various immunoassays. Common analytes include thrombin-AT (coagulation), contact system complexes between proteases and serpins (FXIIa/C1INH, FXIIa/AT, FXIa/C1INH, FXIa/AT, kallikrein/C1INH, kallikrein/AT), and C3a and sC5b-9 (complement). Cytokines and chemokines which are synthesized and released by different populations of leukocytes, as well as by platelets can be analyzed in the plasma by multiplex assay, but this requires longer incubation time (typically up to 4 h).

In order to dissect the mechanisms of activation, specific inhibitors may be added prior to incubation. We use corn trypsin inhibitor (CTI) or lepirudin to inhibit FXII and thrombin, respectively, compstatin and eculizumab to inhibit complement activation at the levels of C3 and C5, respectively, and C5a receptor antagonist (C5aRA) to inhibit C5a mediated cellular activation and signaling [55].

### 4.2 Evaluation of innate immunity activation by NPs in a whole blood model

Using this model in the combination of monitoring with corona formation, we have evaluated the toxicity of TiO_2_ NP that is widely used in various applications, e.g., paint, toothpaste, sunshields, and other personal care products. We demonstrated that the protein corona on these NPs was abundant in most contact activation proteins and that they induce substantial contact system activation already at very low levels (50 ng/mL). In contrast, these NPs did not induce any detectible complement activation [56].

We have also utilized the above-mentioned model to evaluate the toxicity of Fe_2_O_3_ NPs [57] and diesel NP (Ekdahl et al., unpublished results), i.e., two traffic-related species of NPs, which both are similar to the TiO_2_ NPs in size but differ in surface chemistry. Interesting, in both cases, the degree of contact system activation was much lower compared to the previously studied TiO_2_ NP, but they were similarly complement-inert. In both studies, we could detect substantial amounts of pro-inflammatory chemokines and cytokines, and in the case of Fe_2_O_3_ NPs also platelet-derived growth factors [49]. These differences in cytokine profiles were consistent with totally different protein coronas bound to the three types of NPs.

**Figure 3.**
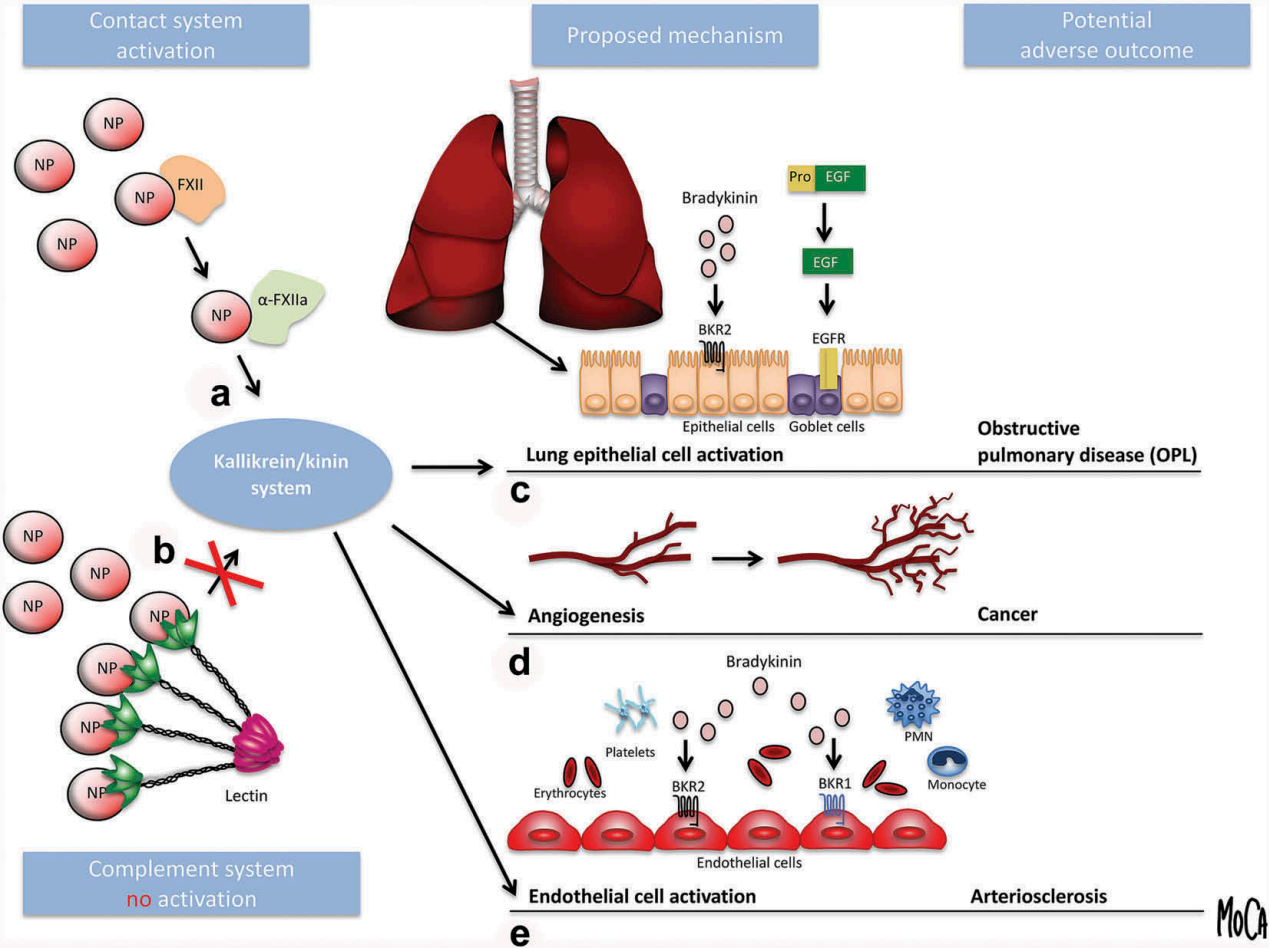
Model of nanoparticle (NP)-induced kallikrein/kinin system activation as a contributing factor in human disease. (A) FXII can get activated to α-FXIIa by direct interaction with NPs thereby initiating the kallikrein/kinin system. (B) The recognition molecules within the complement system (the lectins MBL, ficolins, and collectins within the lectin pathway and C1q within the classical pathway) are all multi-armed molecules which must encounter a conformational change by binding to closely located sites on a surface in order to induce activation of the complement cascade. NPs are too small to harbor more than on binding site and are therefore poor activators of complement. (C) Activated kallikrein can cause lung epithelial cell activation by kinin-dependent and kinin-independent mechanisms, e.g., by the generation of bradykinin and epidermal growth factor (EGF), which bind to their respective receptors (bradykinin receptors 1 and 2, BKR1 and BKR2; EGF receptor, EGFR). Ultimately, this activation may lead to obstructive pulmonary disease (OPD). (D) In addition, bradykinin, which is a potent inducer of angiogenesis, is implicated as a causative agent in cancer [58]. (E) Intravascular inflammation activates the patient’s endothelium, leading to loss of its anti-inflammatory and anti-thrombotic properties and the acquirement of a proinflammatory and prothrombotic phenotype, ultimately resulting in arteriosclerosis. The figure is from ref [57] and reproduced with permission from the publisher.

These studies illustrate the usefulness of our simple and sensitive model and the results have allowed us to construct the model for NP-induced kallikrein/kinin system activation that is depicted in Figure 3. One single FXII can get autoactivated to α-FXIIa by direct interaction with individual NPs thereby initiating the kallikrein/kinin system. In contrast, most recognition molecules within the complement system (the lectins MBL, the ficolins and the collectins within the lectin pathway, as well as C1q within the classical pathway) are all multi-armed molecules which must encounter a conformational change by binding to closely located sites on a surface in order to induce activation of the complement cascade. Typically, NPs are too small to harbor more than on binding site and are therefore poor activators of complement. Kallikrein can cause lung epithelial cell activation by kinin-dependent and kinin-independent mechanisms, e.g.; by the generation of BK and epidermal growth factor (EGF), which bind to their respective receptors: BKR1, BKR2, and the EGF receptor. Ultimately, this activation may lead to obstructive pulmonary disease. Furthermore, BK, which is a potent inducer of angiogenesis, is implicated as a causative agent in cancer [58]. In addition, the patient’s endothelium may get activated by intravascular inflammation, which leads to decay of the glycocalyx and subsequent loss of its anti-inflammatory and anti-thrombotic properties and the acquirement of a proinflammatory and prothrombotic phenotype, ultimately resulting in arteriosclerosis.

## 5. Perspective on the future development of the field of NP toxicity

We find that characterization of the protein corona formed on NPs after incubation in human EDTA-plasma is useful as an initial and fairly rapid screening procedure. Confirmation of the down-stream biological events such as blood cascade system activation and generation and release of various cytokines, chemokines, and growth factors can only be done using whole blood models, which allows to study the extensive amount of interaction between the systems.

Further studies of the toxic effects of nanoparticles on human health are of outmost importance, especially since much of that knowledge still is lacking. However, one of the key challenges is to find a model that is sufficiently complex to generate relevant information about the toxicity of the NP under investigation. Various methods and experimental models are available for nanotoxicity testing. Today the toxicity testing methods are dominated by *in vivo* methods. Although *in vivo* studies allow toxicity testing of pathological changes, biodistribution, and clearance, they tend to be complicated and involves animal experiments that usually are both painful and stressful for the animals. In addition, due to species differences, the results obtained from animal models are of limited use for prediction of human disease. From this aspect, *in vitro* models may be advantageous, as results can be generated faster, at a lower cost, and the ethical issues are avoided. However, the vast majority of *in vitro* nanotoxicity assays used today has large limitations since they examine nanoparticle influence on a single, homogeneous, immortal cell type.

Instead, we propose the development of a new type of human-based *in vitro* model of blood and blood vessels for evaluation of NP triggered pathological events. This system can also be completed with other types of organ-specific cells such as epithelial cells from the lung or the gastrointestinal tract. In contrary to other *in vitro*-systems where only a few purified components are included, all components ranging from the lung cell to the blood vessel are present in our mode, which is designed for native human whole blood. Since all parts of the model system will be exposed to the same particles, the overall toxicity, the cooperativity as well as the specific impact on each element separately can be evaluated.

Abbreviations: EDTA, ethylenediaminetetraacetic acid; PBS, phosphate buffered saline; SPIONS, Superparamagnetic iron oxide nanoparticles

## References

[1] Petri-Fink A, Hofmann H. Superparamagnetic iron oxide nanoparticles (SPIONs): from synthesis to in vivo studies–a summary of the synthesis, characterization, *in**vitro*, and *in**vivo* investigations of SPIONs with particular focus on surface and colloidal properties. IEEE Trans Nanobioscience. 2007;6:289–297. 18217622 10.1109/tnb.2007.908987

[2] Gulumian M, Verbeek J, Andraos C, et al Systematic review of screening and surveillance programs to protect workers from nanomaterials. Ed B Xu PLoS ONE. 2016;11:e0166071. 10.1371/journal.pone.0166071 PMC5102462 27829014

[3] FDI General Assembly Nanoparticles in dental practice: adopted by the FDI General assembly: 7 september 2018, Buenos Aires, Argentina. Int Dent J. 2019;69:23–24. 30697709 10.1111/idj.12472 PMC9379048

[4] Kluger N, Koljonen V Tattoos, inks, and cancer. Lancet Oncol. 2012;13:e161–8. 22469126 10.1016/S1470-2045(11)70340-0

[5] Laux P, Tralau T, Tentschert J, et al A medical-toxicological view of tattooing. Lancet. 2016;387:395–402. 26211826 10.1016/S0140-6736(15)60215-X

[6] Kunzmann A, Andersson B, Thurnherr T, et al Toxicology of engineered nanomaterials: focus on biocompatibility, biodistribution and biodegradation. Biochim Biophys Acta Gen Subj. 2011;1810:361–373. 10.1016/j.bbagen.2010.04.007 20435096

[7] Wilkinson KE, Lundkvist J, Netrval J, et al Space and time resolved monitoring of airborne particulate matter in proximity of a traffic roundabout in Sweden. Environ Pollut. 2013;182:364–370. 23974166 10.1016/j.envpol.2013.07.043

[8] Muala A, Nicklasson H, Boman C, et al Respiratory tract deposition of inhaled wood smoke particles in healthy volunteers. J Aerosol Med Pulm Drug Deliv. 2015;28:237–246. 25393443 10.1089/jamp.2014.1122

[9] Marabini L, Ozgen S, Turacchi S, et al Ultrafine particles (UFPs) from domestic wood stoves: genotoxicity in human lung carcinoma A549 cells. Mutat Res. 2017;820:39–46. 28676265 10.1016/j.mrgentox.2017.06.001

[10] You R, Lu W, Shan M, et al Nanoparticulate carbon black in cigarette smoke induces DNA cleavage and Th17-mediated emphysema. Elife. 2015;4:e09623. 26437452 10.7554/eLife.09623 PMC4612775

[11] Chuang H-C, Jones T, Chen Y, et al Characterisation of airborne particles and associated organic components produced from incense burning. Anal Bioanal Chem. 2011;401:3095–3102. 21769554 10.1007/s00216-011-5209-7

[12] Tepe N, Bau M Importance of nanoparticles and colloids from volcanic ash for riverine transport of trace elements to the ocean: evidence from glacial-fed rivers after the 2010 eruption of Eyjafjallajökull Volcano, Iceland. Sci Total Environ. 2014;488-489:243–251. 24836133 10.1016/j.scitotenv.2014.04.083

[13] Ermolin MS, Fedotov PS, Malik NA, et al Nanoparticles of volcanic ash as a carrier for toxic elements on the global scale. Chemosphere. 2018;200:16–22. 29471164 10.1016/j.chemosphere.2018.02.089

[14] Midander K, Cronholm P, Karlsson HL, et al Surface characteristics, copper release, and toxicity of nano- and micrometer-sized copper and copper(II) oxide particles: a cross-disciplinary study. Small. 2009;5:389–399. 19148889 10.1002/smll.200801220

[15] Nel A, Xia T, Mädler L, et al Toxic potential of materials at the nanolevel. Science. 2006;311:622–627. 16456071 10.1126/science.1114397

[16] Jin X, Ma Q, Sun Z, et al Airborne fine particles induce hematological effects through regulating the crosstalk of the kallikrein-kinin, complement, and coagulation systems. Environ Sci Technol. 2019;53:2840–2851. 30742439 10.1021/acs.est.8b05817

[17] Simkhovich BZ, Kleinman MT, Kloner RA Air pollution and cardiovascular injury epidemiology, toxicology, and mechanisms. J Am Coll Cardiol. 2008;52:719–726. 18718418 10.1016/j.jacc.2008.05.029

[18] Latvala S, Vare D, Karlsson HL, et al *In vitro* genotoxicity of airborne Ni-NP in air-liquid interface. J Appl Toxicol. 2017;37:1420–1427. 28815640 10.1002/jat.3510 PMC5697686

[19] Latvala S, Hedberg J, Möller L, et al Optimization of an air-liquid interface exposure system for assessing toxicity of airborne nanoparticles. J Appl Toxicol. 2016;36:1294–1301. 26935862 10.1002/jat.3304 PMC5069579

[20] Sirisinha S Evolutionary insights into the origin of innate and adaptive immune systems: different shades of grey. Asian Pac J Allergy Immunol. 2014;32:3–15. 24641285

[21] Boehm T, Swann JB Origin and evolution of adaptive immunity. Annu Rev Anim Biosci. 2014;2:259–283. 25384143 10.1146/annurev-animal-022513-114201

[22] Ekdahl KN, Teramura Y, Hamad OA, et al Dangerous liaisons: complement, coagulation, and kallikrein/kinin cross-talk act as a linchpin in the events leading to thromboinflammation. Immunol Rev. 2016;274:245–269. 27782319 10.1111/imr.12471

[23] Yang G, Lucas R, Caldwell R, et al Novel mechanisms of endothelial dysfunction in diabetes. J Cardiovasc Dis Res. 2010;1:59–63. 20877687 10.4103/0975-3583.64432 PMC2945199

[24] Andersson J, Larsson R, Richter R, et al Binding of a model regulator of complement activation (RCA) to a biomaterial surface: surface-bound factor H inhibits complement activation. Biomaterials. 2001;22:2435–2443. 11511041 10.1016/s0142-9612(00)00431-2

[25] Nilsson B, Ekdahl KN, Mollnes TE, et al The role of complement in biomaterial-induced inflammation. Mol Immunol. 2007;44:82–94. 16905192 10.1016/j.molimm.2006.06.020

[26] Horbett TA Chapter 13 Principles underlying the role of adsorbed plasma proteins in blood interactions with foreign materials. Cardiovasc Pathol. 1993;2:137–148.25990608

[27] Nakanishi K, Sakiyama T, Imamura K On the adsorption of proteins on solid surfaces, a common but very complicated phenomenon. J Biosci Bioeng. 2001;91:233–244. 16232982 10.1263/jbb.91.233

[28] Andersson J, Ekdahl KN, Larsson R, et al C3 adsorbed to a polymer surface can form an initiating alternative pathway convertase. J Immunol. 2002;168:5786–5791. 12023380 10.4049/jimmunol.168.11.5786

[29] Tengvall P, Askendal A, Lundström I Complement activation by IgG immobilized on methylated silicon. J Biomed Mater Res. 1996;31:305–312. 8806055 10.1002/(SICI)1097-4636(199607)31:3<305::AID-JBM3>3.0.CO;2-Q

[30] Vogler EA, Siedlecki CA Contact activation of blood-plasma coagulation. Biomaterials. 2009;30:1857–1869. 19168215 10.1016/j.biomaterials.2008.12.041 PMC2705825

[31] Hulander M, Lundgren A, Faxalv L, et al Gradients in surface nanotopography used to study platelet adhesion and activation. Colloids Surf B Biointerfaces. 2013;110:261–269. 23732803 10.1016/j.colsurfb.2013.04.010

[32] Zhuo R, Siedlecki CA, Vogler EA Autoactivation of blood factor XII at hydrophilic and hydrophobic surfaces. Biomaterials. 2006;27:4325–4332. 16644008 10.1016/j.biomaterials.2006.04.001

[33] Zhuo R, Siedlecki CA, Vogler EA Competitive-protein adsorption in contact activation of blood factor XII. Biomaterials. 2007;28:4355–4369. 17644174 10.1016/j.biomaterials.2007.06.019 PMC2705829

[34] Wu Y Contact pathway of coagulation and inflammation. Thromb J. 2015;13:17. 25949215 10.1186/s12959-015-0048-y PMC4421925

[35] Golias C, Charalabopoulos A, Stagikas D, et al The kinin system–bradykinin: biological effects and clinical implications. Multiple role of the kinin system–bradykinin. Hippokratia. 2007;11:124–128. 19582206 PMC2658795

[36] O’Toole TE, Conklin DJ, Bhatnagar A Environmental risk factors for heart disease. Rev Environ Health. 2008;23:167–202. 19119685 10.1515/reveh.2008.23.3.167

[37] Triantafilou K, Hughes TR, Triantafilou M, et al The complement membrane attack complex triggers intracellular Ca^2+^ fluxes leading to NLRP3 inflammasome activation. J Cell Sci. 2013;126:2903–2913. 23613465 10.1242/jcs.124388

[38] Tedesco F, Pausa M, Nardon E, et al The cytolytically inactive terminal complement complex activates endothelial cells to express adhesion molecules and tissue factor procoagulant activity. J Exp Med. 1997;185:1619–1627. 9151899 10.1084/jem.185.9.1619 PMC2196305

[39] De Matteis V, Rinaldi R Toxicity Assessment in the Nanoparticle Era. Adv Exp Med Biol. 2018;1048:1–19. 29453529 10.1007/978-3-319-72041-8_1

[40] Ricklin D, Hajishengallis G, Yang K, et al Complement: a key system for immune surveillance and homeostasis. Nat Immunol. 2010;11:785–797. 20720586 10.1038/ni.1923 PMC2924908

[41] Hulander M, Lundgren A, Berglin M, et al Immune complement activation is attenuated by surface nanotopography. Ijn. 2011;6:2653. 22114496 10.2147/IJN.S24578 PMC3218579

[42] Sanfins E, Augustsson C, Dahlbäck B, et al Size-dependent effects of nanoparticles on enzymes in the blood coagulation cascade. Nano Lett. 2014;14:4736–4744. 25025946 10.1021/nl501863u

[43] Kushida T, Saha K, Subramani C, et al Effect of nano-scale curvature on the intrinsic blood coagulation system. Nanoscale. 2014;6:14484–14487. 25341004 10.1039/c4nr04128c PMC4224616

[44] Monopoli MP, Aberg C, Salvati A, et al Biomolecular coronas provide the biological identity of nanosized materials. Nat Publishing Group. 2012;7:779–786. 10.1038/nnano.2012.207 23212421

[45] Lynch I, Cedervall T, Lundqvist M, et al The nanoparticle-protein complex as a biological entity; a complex fluids and surface science challenge for the 21st century. Adv Colloid Interface Sci. 2007;134-135:167–174. 17574200 10.1016/j.cis.2007.04.021

[46] Lundqvist M, Augustsson C, Lilja M, et al The nanoparticle protein corona formed in human blood or human blood fractions. ed S Hussain. PLoS ONE. 2017;12:e0175871. 28414772 10.1371/journal.pone.0175871 PMC5393619

[47] Kelly PM, Aberg C, Polo E, et al Mapping protein binding sites on the biomolecular corona of nanoparticles. Nat Publishing Group. 2015;10:472–479. 10.1038/nnano.2015.47 25822932

[48] Pearson RM, Juettner VV, Hong S Biomolecular corona on nanoparticles: a survey of recent literature and its implications in targeted drug delivery. Front Chem. 2014;2:108. 25506050 10.3389/fchem.2014.00108 PMC4245918

[49] Hu Z, Zhao L, Zhang H, et al The on-bead digestion of protein corona on nanoparticles by trypsin immobilized on the magnetic nanoparticle. J Chromatogr A. 2014;1334:55–63. 24572545 10.1016/j.chroma.2014.01.077

[50] Vogt C, Pernemalm M, Kohonen P, et al Proteomics analysis reveals distinct corona composition on magnetic nanoparticles with different surface coatings: implications for interactions with primary human macrophages. ed V Bansal. PLoS ONE. 2015;10:e0129008. 26444829 10.1371/journal.pone.0129008 PMC4596693

[51] Sakulkhu U, Maurizi L, Mahmoudi M, et al Ex situ evaluation of the composition of protein corona of intravenously injected superparamagnetic nanoparticles in rats. Nanoscale. 2014;6:11439–11450. 25154771 10.1039/c4nr02793k

[52] Quigley JD, Drewry JJ Nutrient and immunity transfer from cow to calf pre- and postcalving. J Dairy Sci. 1998;81:2779–2790. 9812284 10.3168/jds.S0022-0302(98)75836-9

[53] Sakulkhu U, Mahmoudi M, Maurizi L, et al Protein corona composition of superparamagnetic iron oxide nanoparticles with various physico-chemical properties and coatings. Sci Rep. 2014;4:5020. 24846348 10.1038/srep05020 PMC5381372

[54] Khan S, Ansari AA, Rolfo C, et al Evaluation of in vitro cytotoxicity, biocompatibility, and changes in the expression of apoptosis regulatory proteins induced by cerium oxide nanocrystals. Sci Technol Adv Mater. 2017;18:364–373. 28634498 10.1080/14686996.2017.1319731 PMC5468938

[55] Ekdahl KN, Hong J, Hamad OA, et al Evaluation of the blood compatibility of materials, cells, and tissues: basic concepts, test models, and practical guidelines. Adv Exp Med Biol. 2013;735:257–270. 23402033 10.1007/978-1-4614-4118-2_18

[56] Ekstrand-Hammarström B, Hong J, Davoodpour P, et al TiO_2_ nanoparticles tested in a novel screening whole human blood model of toxicity trigger adverse activation of the kallikrein system at low concentrations. Biomaterials. 2015;51:58–68. 25770998 10.1016/j.biomaterials.2015.01.031

[57] Ekdahl KN, Davoodpour P, Ekstrand-Hammarström B, et al Contact (kallikrein/kinin) system activation in whole human blood induced by low concentrations of α-Fe_2_O_3_ nanoparticles. Nanomedicine. 2018;14:735–744. 29277639 10.1016/j.nano.2017.12.008

[58] Da Costa PLN, Sirois P, Tannock IF, et al The role of kinin receptors in cancer and therapeutic opportunities. Cancer Lett. 2014;345:27–38. 24333733 10.1016/j.canlet.2013.12.009

[59] Hu Z, Zhang H, Zhang Y, et al Nanoparticle size matters in the formation of plasma protein coronas on Fe_3_O_4_ nanoparticles. Colloids Surf B Biointerfaces. 2014;121:354–361. 24974013 10.1016/j.colsurfb.2014.06.016

[60] Landgraf L, Christner C, Storck W, et al A plasma protein corona enhances the biocompatibility of Au@Fe_3_O_4_ Janus particles. Biomaterials. 2015;68:77–88. 26276693 10.1016/j.biomaterials.2015.07.049

[61] Klapper Y, Hamad OA, Teramura Y, et al Mediation of a non-proteolytic activation of complement component C3 by phospholipid vesicles. Biomaterials. 2014;35:3688–3696. 24462362 10.1016/j.biomaterials.2013.12.085 PMC4104820

